# A systems biology approach to invasive behavior: comparing cancer metastasis and suburban sprawl development

**DOI:** 10.1186/1756-0500-3-36

**Published:** 2010-02-10

**Authors:** John J Ryan, Benjamin L Dows, Michael V Kirk, Xueming Chen, Jeffrey R Eastman, Rodney J Dyer, Lemont B Kier

**Affiliations:** 1 Department of Biology, Virginia Commonwealth University, Richmond, VA 23284, USA; 2 Center for the Study of Biological Complexity, Virginia Commonwealth University, Richmond, VA 23284, USA; 3 Urban and Regional Planning Program, L. Douglas Wilder School of Government and Public Affairs, Virginia Commonwealth University, Richmond, VA 23284, USA; 4 Department of Community Development, City of Richmond, VA 23220, USA

## Abstract

**Background:**

Despite constant progress, cancer remains the second leading cause of death in the United States. The ability of tumors to metastasize is central to this dilemma, as many studies demonstrate successful treatment correlating to diagnosis prior to cancer spread. Hence a better understanding of cancer invasiveness and metastasis could provide critical insight.

**Presentation of the hypothesis:**

We hypothesize that a systems biology-based comparison of cancer invasiveness and suburban sprawl will reveal similarities that are instructive.

**Testing the hypothesis:**

We compare the structure and behavior of invasive cancer to suburban sprawl development. While these two systems differ vastly in dimension, they appear to adhere to scale-invariant laws consistent with invasive behavior in general. We demonstrate that cancer and sprawl have striking similarities in their natural history, initiating factors, patterns of invasion, vessel distribution and even methods of causing death.

**Implications of the hypothesis:**

We propose that metastatic cancer and suburban sprawl provide striking analogs in invasive behavior, to the extent that conclusions from one system could be predictive of behavior in the other. We suggest ways in which this model could be used to advance our understanding of cancer biology and treatment.

## Background

Cancer is a growing problem in developed nations, and remains the second leading cause of death in the United States. While constant progress is being made, improvements in survival have come more slowly than desired. In the past three decades the five-year survival rate for all cancers combined has risen from 50% to 66% in the US. While the death rates for cancers of the lung, colon, breast, and prostate have improved in the past twenty years, there has been little change in death caused by pancreatic, ovarian, or liver cancer [1]. Although cancer is a collection of distinct neoplasias, all share the attribute of invasiveness. This hallmark is perhaps the most important aspect of cancer needing new insight, since cancers that have not metastasized generally show higher cure rates [1].

The invasive nature of cancer is an acquired trait that develops as neoplasias undergo a process of selective expansion over a period of years [2,3]. The inappropriate expression of genes empowering cells with movement, survival, and angiogenesis capacity is accomplished through cumulative mutations and gene de-repression, yielding cellular clones able to cause systemic disease. To understand cancer metastasis, a focus on how these acquired traits are developed and their effects on disease resilience would be informative. A system offering new insights into this process is one desirable means of accomplishing this goal.

Modeling complex processes by mathematical means or by comparisons to systems with known behaviors has proven to be an effective approach in gaining insight. Striking examples of this application include the planetary model of the atom, which is still employed today. Cancer has been postulated to behave in ways consistent with evolutionary processes since the 1970s [3,4]. Merlo and co-workers recently described how both evolutionary and ecological laws might be used to model cancer to develop new insight, making several interesting speculations, including the observation that pulsatile chemotherapy could be less efficacious than lower dose chronic therapy [4]. Likewise, Marco and co-workers have found that glioma metastasis behaves with striking similarity to the spread of *Ulmus procera*, the English elm, an invasive species found in Argentinian forests [5]. These models support the development of new ways to understand how cancer spreads.

While biological models such as invasive species offer new interpretations, there are inherent limits to their use, since our understanding of their behavior is also developing. Mathematical models of artificial systems have the opposite constraint of needing to be tested to determine their robustness in a viable biological system with unexpected variables. For these reasons it would be beneficial to examine a system bearing strong similarities to metastasis but following laws that are well described. A suitable model would match metastatic cancer in the characteristics of structure, initiating factors, invasiveness, clonal expansion, angiogenesis, and patient death.

## Presentation of the Hypothesis

We propose that a useful model for gaining novel insight about invasive behavior is the growth of suburban areas, specifically those fitting what has commonly been termed "sprawl" development. These two systems differ tremendously in scale, but represent an analogous pair, as we detail below. They may follow scale-invariant laws related to invasive behavior.

## Testing the Hypothesis

### Structural Similarities

**Figure 1:**
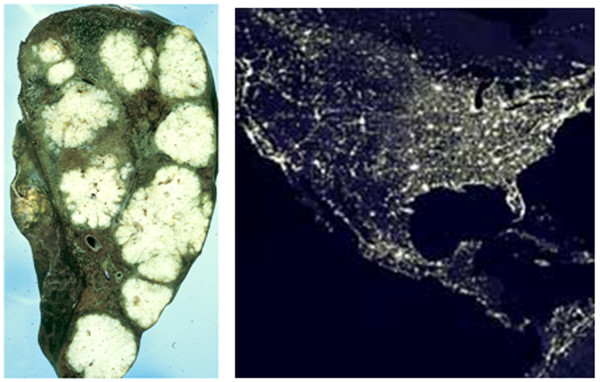
**Structural Similarities**. The figure on left shows multiple tumors derived from colon cancer that has metastasized to the liver. The right shows nighttime imaging of the United States. Both structures demonstrate a diffuse, fractal-like structure of invasiveness. Satellite image is courtesy of the Image Science and Analysis Laboratory, NASA Johnson Space Center. Liver section image is courtesy of Peter Isaacson, University College, London.

The most simplistic observation is readily detected by comparisons of histological examination of cancer versus satellite imaging of city growth (Figure 1). Suburban sprawl and malignant cancers both display an invasive style of growth, employing local and long-distance mechanisms. In fact the growth of both systems has been shown to fit the mathematical description of a fractal pattern [6-14], though the two structures have not been directly compared. An important aspect of this comparison is that sprawl growth, while having emergent properties, is largely dictated by documented man-made laws. There have also been many attempts, some successful, in limiting or preventing sprawl growth. If this matched pair fits the tenets of general systems theory, it is plausible that suburban sprawl can yield insights into cancer not easily obtained from biological systems.

The growth pattern of cancer and sprawl can be quantified by fractal analysis, yielding a fractal dimension value. Fractal dimensionality, *D*, can be measured using several different techniques. For image data, the most common of these is the box method [15]. The box method simply divides the complete image using a grid whose box size is denoted as *h*. For each box size imposed over the image, one counts the number of cells (*N*_*h*_) that are filled with the feature of interest (e.g,. sprawl or cancer). Dimensionality is estimated as *D *= -lim_h → 0 _log (*N*_*h*_)/log (*h*), which corresponds to the slope of the plot of the number of filled boxes versus box size. As an example, a 1-dimensional line whose image has been divided into a grid of 20 × 20 would have a dimensionality *D *= -log(20)/log(1/20) = 1 whereas a box covering the entire image would have a dimensionality of *D *= -log(400)/log(1/20) = 2. What the fractal dimensionality parameter provides is a statistical estimate of how an object fills a space across a broad range of scales.

While fractal measurements offer only initial support for similarity, systems with similar behaviors tend to produce fractals with similar dimension values, making this a logical screening tool for relationships between systems. In support of their similar appearances, the fractal dimension values for cancer and suburban sprawl are closely matched. Ovarian cancer and glioma have been calculated to have *D *values between 1.20-1.38 [16,17], while sprawl growth had *D *= 1.23-1.31 in a recent study ([18]; and personal correspondence). Other analogous systems suggested as cancer models are not as closely matched. For example, Marco and co-workers found invasive elm dispersal to have higher *D *values of 1.6-1.7 [16]. These more closely match the *D *values for traditional urban areas, which range from 1.5-1.8 [19] and have also been suggested to resemble malignant neoplasms [20].

These differences in fractal dimension among possible analogous systems may offer insight into behavior. The predicted power law used to calculate *D *values indicated that for elm dispersal, invasiveness has few constraints related to "field details", essentially meaning that the environment in which the elm tree is located does not greatly influence its ability to spread [16]. This conclusion is consistent with a wind-dispersed invasive species. By comparison, the smaller *D *value for gliomas, calculated in the same study, was consistent with more constrained invasiveness [16]. The physical constraints on cancer metastasis, which include connective tissue hindrance, access to blood or lymphatic vessels, and requirements for angiogenesis, mirror the limitations on suburbanization of undeveloped areas, which can differ from traditional urban development. Issues of geography, road access, and urban services can limit sprawl growth. These similarities suggest that the structural homology of metastasis and sprawl are more than coincidental, and may result from scale-invariant behaviors of an analogous pair. The closely matched fractal dimension measurements further indicate that sprawl-styled growth is, at least in mathematical terms, a plausible model for cancer invasiveness.

### Similar Natural Histories

Both cancer and sprawl represent a new form of growth in a larger "organism". In the case of a neoplasm or "new growth", the form and behavior of the tumor cells is novel to the individual. Invasiveness is noted in many cells, such as the movement of immune cells mobilized to fight infection. However, the widely disseminated movement and continued proliferation of cancer cells is unmatched by any non-pathological process in an adult. Tumors are also unusual in that they expand as a clone of incompletely developed cells with abnormal properties including prolonged survival and dysregulated proliferation. Cancer is truly a qualitatively new form of growth in the body's natural history.

The same can be said for suburban sprawl. Until the 1950's, urbanization followed a centuries-old centric pattern, slowly expanding around the borders of existing cities. Further, the type of development was always mixed, including a variety of land uses. The departure from this style of development was stark and rapid in the United States, as federal loan programs funded construction of 11 million new homes shortly after World War II. This transition was also remarkable in form. Since loan programs specified use of money for newly constructed houses, whole tracts of land were developed for single-use residential housing for the first time, leading to the secondary growth of single-use strip-mall style shopping centers. These segregated structures frequently were constructed from a limited set of blueprints, leading to something akin to clonal expansion. This segregated low-density structure was a new development in the world's natural history (discussed in detail by Duany and co-workers [21]).

### Analogous Initiating Factors

**Figure 2:**
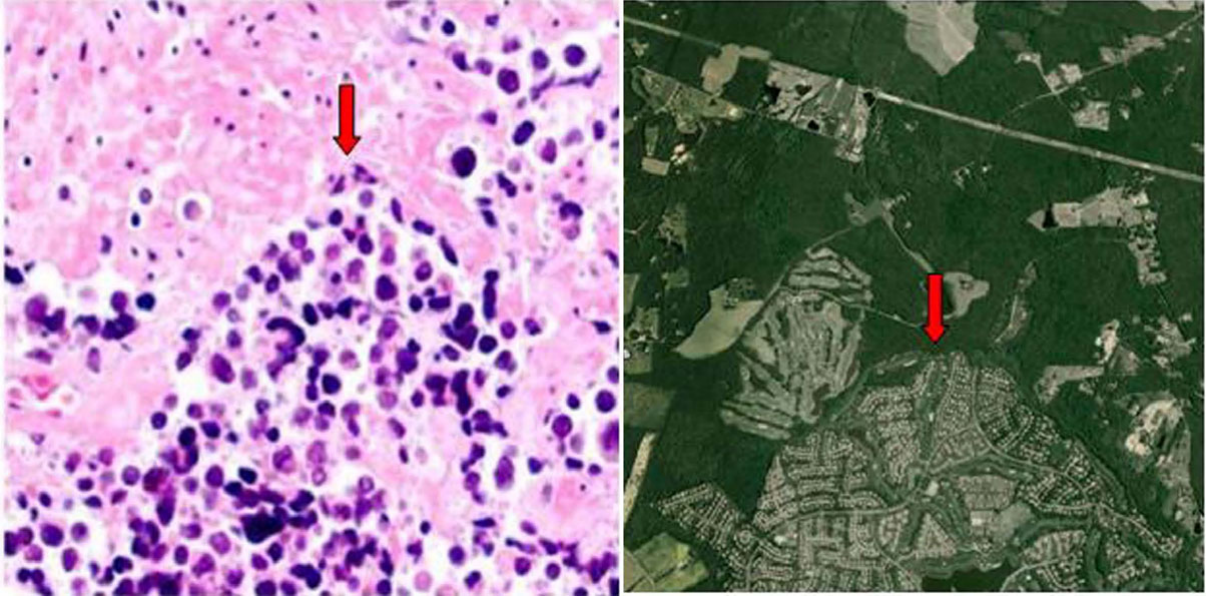
**Similar Initiating Factors**. Left side figure shows invasive bladder carcinoma (arrow) invading into normal tissue. These tumors are often induced by the overproduction of growth-promoting hormones. Right side shows aerial imaging of residential and retail development (arrow) encroaching into a rural area. These developments are most often initiated by economic potential rather than societal need. Satellite image courtesy of Google 2008 Tele Atlas; photomicrograph courtesy of William Frable, M.D., used with permission.

A central question receiving much attention in cancer biology is how tumors begin. Years of work in this area has yielded precise biological answers with analogies to sprawl-styled development. Since the 1970's, Knudson's "two-hit hypothesis" [22] has suggested that most tumors develop slowly over a period of years by accumulating mutations. While the specific genetic changes vary between tumor types, a common theme exists. First, most tumors gain the propensity to proliferate due to an overabundance of growth factors or due to alterations in the cellular signaling pathways that convey growth factor signals to the cell. In either case, the developing cancer cells are "instructed" to proliferate continuously. Second, a majority of cancer cells lose the function of key inhibitory molecules that should suppress proliferation and instruct cells to die. The most commonly mutated tumor suppressor, p53, is functionally absent in 2 of every 3 cancers [23,24]. These two changes allow the development of cancer clones. A sub-group of these cells expresses tissue-degrading proteases and angiogenic factors, allowing for invasive growth (Figure 2).

New anti-cancer drug therapies take advantage of these changes. For example, the effective chemotherapies Tamoxifen and Gleevec block growth factors or intracellular signals inducing proliferation, respectively, that induce proliferation [25,26]. Experimental therapies are targeting matrix metalloproteinases required for invasion, as well as the angiogenic factors stimulating capillary growth [27,28]. However, p53 dysfunction has proven to be a difficult barrier to standard chemotherapy, as p53-null tumors comprise a large percentage of recurrent, drug-resistant cancers [29]. Perhaps a model system could point the way to new ideas for attacking this problem.

**Figure 3:**
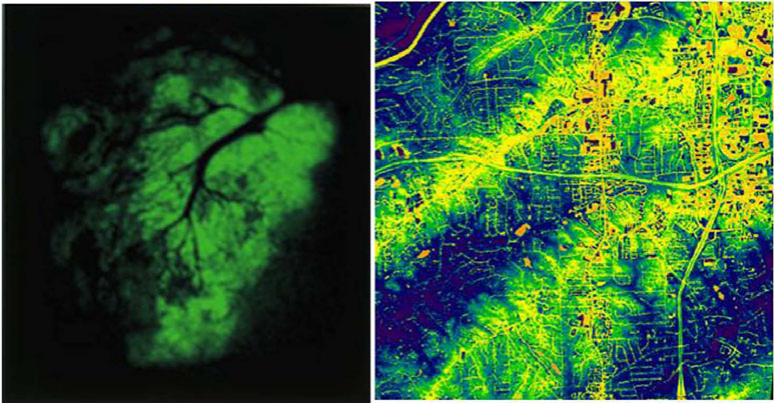
**Similar Patterns of Invasiveness**. Left figure demonstrates dispersion of tumor cells into lung tissue, along existing blood and lymphatic vessels. Right figure shows the prominence of roadways in sprawl development, illustrated by heat dissipation around dwellings and roads. Satellite image is courtesy of the Image Science and Analysis Laboratory, NASA Johnson Space Center. Tumor imaging reproduced from [66], used with permission.

Suburban development has become stunningly invasive, consuming an amount of land that is difficult to contemplate (Figure 2). For example, from 1970-1990 the population of Los Angeles increased 45%, while its land mass grew by 300%. The United States currently develops 7000 acres of land every day, paving an area the size of Delaware each year ([30], p.12). Like cancer, this form of growth is clearly not sustainable. The stimulating factors for sprawl-style growth are strikingly analogous to tumor formation. The original VA and FHA loans made after World War II restricted funds to new residential housing, greatly limiting the diversity of development ([31], pp 205-208). In recent years, suburban sprawl has largely been driven by financial speculation rather than actual need - a fact that has left these developments sensitive to shifts in the economy [32] (Figure 3). This style of land use is, like cancer, lacking in diversity and highly invasive.

There are obvious analogies in these two systems. The growth factors that elicit tumor formation are selective, inducing the expansion of specific populations, such that the outcome is a limited array of cells that eventually become clonal. It is intuitive that blocking growth factors can suppress tumor formation. However, years of attempts to limit suburbanization may offer more insight. Portland, Oregon is a representative case study of both successes and failures. Since 1979 the Portland metropolitan area has had strict controls on urban growth, formally delineated by an Urban Growth Boundary (UGB) designed to protect land from suburban sprawl and promote use of public transit. The success of this fixed boundary has been debated. Between 1990 and 2000, auto share within the Portland UGB decreased modestly from 87.9% to 85.5%, while transit share increased slightly from 7.2% to 9.0% [33]. However, 98% of the population relocating to the Portland metropolitan area from 2000-2004 moved to residences outside of the designated UGB [34]. There appears to be sufficient financial impetus created by higher housing costs or other issues within the UBG to promote growth outside of the designated area. This case study illustrates the difficulty in completely blocking the initiating factors for sprawl as well as the promise and incremental gains they are achieving. Tumors often escape the suppressive effects of growth factor inhibitors, such as the HER2/neu antagonist Herceptin [35]. One wonders if these two systems share similar challenges and if one system might predict success or failure in another.

If financial profit is analogous to growth factor signaling, a viable comparison to the normally suppressive actions of p53 may be gasoline. A survey of ten U.S. cities showed that gasoline consumption is significantly related to gasoline price (-.6151) and vehicle ownership (+.6574) [36]. It is well documented in both America and Europe that high-density land use reduces the need for automobile travel also limits gasoline consumption [37,38]. Hence a natural barrier to sprawl should be transportation costs. However, gasoline has been significantly cheaper in the United States compared to countries where sprawl-style growth is uncommon. Gordon and Richardson noted that the inflation-adjusted cost of gasoline over a 20 year period decreased in the U.S., encouraging households to substitute housing costs for transportation costs by moving farther from cities, thus increasing suburbanization [39]. With sprawl development increasing, Americans drive twice as many miles today as they did 20 years ago. In fact, the number of car miles driven has grown at 4 times the population rate since 1969 ([40], p.15).

The low cost of auto travel in the U.S. has led some to refer to it as a "free good" in economics terms [41]. Rather than beneficial, free goods are generally destabilizing, as they increase demand in a manner that is unpredictable. In these respects, gasoline costs and p53 are analogous. The expression of p53 is supposed to represent a natural barrier to tumor growth, so its absence in tumors leads these cells to behave abnormally, ignoring normal limits on proliferation. There is much interest in restoring p53 expression to tumor cells, however the outcome of this remains unknown. With recent fluctuations in the price of gasoline, it may be possible to discern the effects on suburbanization. Since gasoline cost and consumption have been inversely correlated, higher fuel costs are predicted to slow suburbanization. In keeping with the tenets of systems biology, we may be able to predict the effects of modulating p53 in tumors based upon observations made in the realm of suburbanization and changes in transportation costs.

### Similar Patterns of Invasion

Tumor invasion proceeds by predictable steps to form local or distant metastases. Carcinomas, the most common kind of cancer, must first detach from their surrounding cells, degrade the connective tissues around them, and invade into an existing lymphatic or blood vessel before migrating to new sites of growth (Figure 3). Once arriving at a new location, cancer cells must induce the growth of new blood and lymphatic vessels into the newly forming secondary tumor, literally creating a new local circulatory system. This process requires tumor cells to possess abilities not found in their normal counterparts. First, cancer cells ignore normal constraints on cell growth, even resisting the powerful inhibitory signals halting proliferation when DNA is damaged. Eventually a clone or group of clones is derived that possesses long term survival and proliferation capabilities, supported by the infrastructure of blood vessels necessary to exchange gases and remove waste products.

Sprawl-style growth follows a strikingly similar pattern, with both local and distant invasion. Sprawl development follows existing travel corridors, but quickly requires new roads, utility services, schools and other municipal support. This also yields further development, much like secondary tumors yield further metastases. While localized spread is often seen, so-called "leap-frog" development skirts boundaries or barriers, allowing distant growth of previously undeveloped land. The continued survival and expansion of sprawl communities is dependent upon infrastructural support, and tends to ignore normally powerful constraints on growth. For example, the cost of one mile of interstate highway is estimated at $30 million ([30], p.121), with schools and municipal services costing much more. Yet these public asset-consuming communities not only survive; they are the predominant form of development in the United States over the past half-century.

### Similar Clonal Expansion

**Figure 4:**
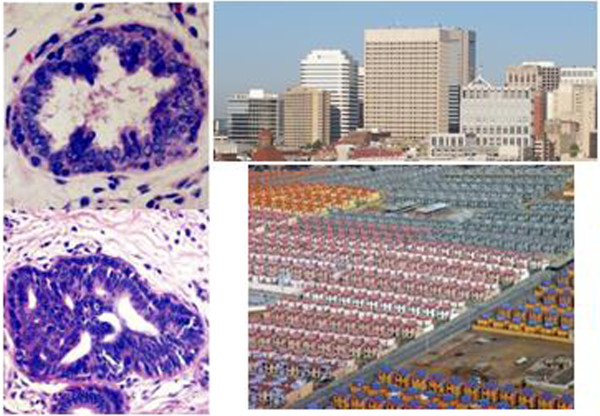
**Similar Patterns of Clonal Expansion**. Left side shows normal mammary duct and surrounding differentiated tissues (top), compared to pre-cancerous hyperplastic ductal tissue with prominent glandular duct cells staining dark blue (bottom) (adapted from *Atlas of Diagnostic Oncology*, edited by Arthur Skarin; used with permission. Right side shows a differentiated urban area with various forms of residential, retail, and civic structures, compared to lower photo of suburban residential dwellings (top photo by John Ryan; bottom courtesy of Carlos Oscar Ruiz, used with permission).

Organs and tissues possess cellular diversity required for proper functioning. Loss of this diversity is obvious in hyperplastic states that increase the number of one group of cells. This can be a precursor to cancer, a group of still-normal cells expanding out of proportion to their environment, destroying the diversity needed for long term survival of the organ (Figure 4).

As stated, cancer spreads throughout the body in a clonal fashion, generated from a small group of founder cells derived from the primary tumor. This form of invasion is noteworthy not just from the novel aspect of so many cells coming from one source. In fact, the drug-resistant, life-ending population of cells that eventually kills the patient is thought to be present at an early stage of cancer's dissemination, perhaps propagated by indigenous stem cells [42]. This small population possesses some genetically-encoded ability to resist cytotoxic chemotherapy, and expands as a drug-resistant group often detected only after cancer recurs. It is this clonal expansion, and our inability to detect this dangerous subpopulation early in the disease that matters so very much to the patient. Ample evidence suggests that finding and killing these cells would improve clinical outcomes.

Suburban sprawl is notable not only for its scale, but for a striking sameness in design. Some suburban streets possess rows of houses differing little beyond paint color (Figure 4). Office parks, retail space and even public buildings such as schools have been developed with extremely limited diversity. The lack of building variety in sprawl is amplified by zoning law constraints dictating segregation of incompatible uses. These zoning laws not only predict sprawl-style development; they proscribe it. For example, the acres of parking, distant set-back positions from roads, lack of on-street parking, and physical separation of retail, office, and residential buildings are all required by law. The result of these zoning constraints has been profound, yielding the now-familiar housing developments, office parks, and strip malls - each of which requires vehicle transport for access. This stands in stark difference to traditional towns, where one could live, work, and dine, attend school, religious, and civic events all in walking distance. The buildings of compact towns mirror their divergent uses, with a variety of styles found even within a limited number of architectural types. While cancer represents the true definition of a monoculture - consisting of a single cell type, nearly all of which are genetically identical - suburban housing is not far off the mark.

Growth in the form of sprawl must offer advantages. It is important to note that 80% of all structures built in America were constructed since the 1950's [43]. This is especially true in suburban areas, where a combination of large-scale interstate highway construction, near-universal auto ownership, advanced telecommunication technology, favorable federal housing policies, inexpensive suburban land, and better suburban school quality, have made central city's geographic location less important. In addition, Many American households, especially middle and upper class families, have shown a preference for the suburban lifestyle. Reasons include a preference towards lower-density development (for lower ambient noise and increased privacy), better schools, less crime, and a generally slower lifestyle than the urban one [39]. Therefore, before reaching its turning point to show negative consequences, this suburban sprawl model seems to be a rational choice for many people.

So too with cancer growth - once an aggressive clone of cells has developed through its latency, a huge number of cells, literally hundreds of billions, can be produced in a period of months. Perhaps cancer and sprawl are similarly successful because of their simplicity of design. Cancer cells can lose many of the markings of the tissue from which they originated, becoming *anaplastic*, or without form. In place of the genes that gave them a specified function in the body, these cells now have a narrowed genetic focus on continued proliferation. In fact many cancer cells are quite fragile, susceptible to a variety of cell-killing insults that minimally damage normal cells. It is this fact, coupled with the constantly proliferating nature of tumors that allows chemotherapy to work successfully [44]. Sprawl-styled development is both rapid and relatively low-cost. In some cases this style of building has led to questions of construction quality in addition to the lack of defining features to set apart one structure from another ([30], pp.39-42). These features of vast clonal expansion, coupled with the presence of rapidly-produced but undifferentiated, poorly constructed products suggest that cancer and sprawl share not only appearances but behaviors as well.

There is much interest in promoting cell differentiation to suppress cancer development. For example, the drug 5-aza-2'-deoxycytidine reverses DNA hypomethylation and can induce differentiation in some cancers [45]. This concept of promoting differentiation may be furthered by comparisons to suburbanization. There have been many attempts to promote diversity in suburban areas, including the building of entire communities as small towns - so-called "greenfield" developments. Even though the impact of this development model on commuting and land use has been debated [46], it may offer some insight into how differentiation as a mode of therapy might be best explored.

One good example of promoting differentiation and diversity in suburban development is the Rosslyn-Ballston Corridor in Arlington County, Virginia, which is a suburban county of Washington, D.C. The Washington metropolitan area has seen tremendous suburban growth, expanding at the rate of 22 km^2^/year from 1973-1996 [47] and eliciting interest in ways to curb sprawl. The Rosslyn-Ballston Corridor encompasses high-density, diverse, and mixed-use transit-oriented developments centered around five public transit stations. As shown in Table 1, this Corridor and the similar Jefferson-Davis Corridor have achieved remarkable successes in reducing auto use while promoting transit use, which are important to realizing a sprawl-free "Greenfield" development. It is interesting to note that the rates of auto drivers and transit use in the designated Corridors is strikingly similar to those in the District of Columbia, an area that has highly-differentiated land use and architecture. In contrast, nearby Loudon and Prince William counties, which lack the "differentiated" nature of the corridors, have approximately 50% higher rates of auto drivers and 90% less transit use. These observations support the concept that differentiation can suppress some of the negative consequences of invasiveness.

**Table 1:** Greater Washington Metropolitan Region Mode Splits in June 2009

Transit Mode	District of Columbia	Arlington County			Loudoun County	Prince William County
				
		County Total	Rosslyn-Ballston Corridor	Jefferson Davis Corridor		
Auto Driver	37.0	52.4	45.0	36.0	63.3	59.7
Auto Passenger	14.4	17.8	11.0	17.0	26.4	28.9
Transit	18.3	10.7	19.0	19.0	1.3	2.2
Walk/Bike	27.5	16.0	22.0	25.0	3.9	4.1
School Bus/Other	2.5	3.2	3.0	4.0	5.2	5.1

Total	99.7	100.0	101.0	100.1	100.0	100.0

Source: Greater Washington Metropolitan Region 2007/2008 Household Transportation Survey.

### Analogous "Vessel" Distribution Systems

**Figure 5:**
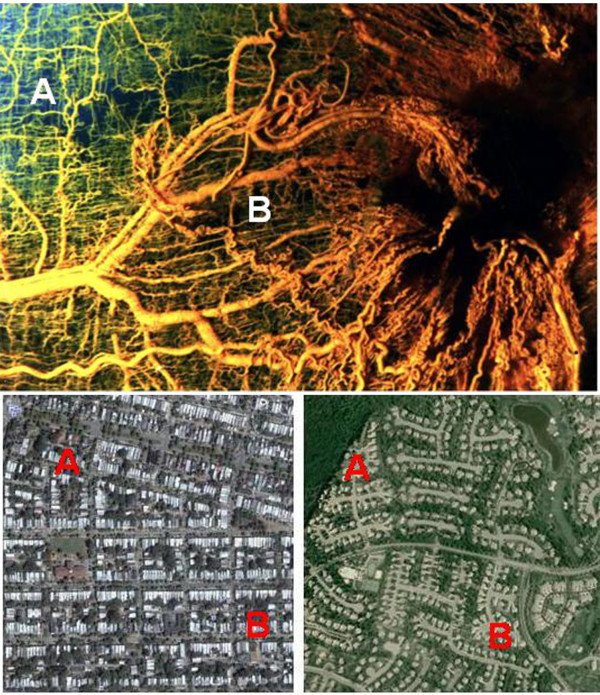
**Similar Patterns of Inefficient "Vessel" Formation**. Top shows normal (A) versus tumor-elicited (B) blood vessel pattern, illustrating differences in design efficiency. Photomicrograph by L. Heuser and R. Ackland, University of Louisville School of Medicine. Bottom shows traditional development with street grid pattern, compared to sprawl development. The possible pathways and distance from location "A" to location "B" is indicative of differences in distribution efficiency. Satellite images courtesy of Google 2008 Tele Atlas.

Tumor angiogenesis is absolutely critical for tumor invasion and metastasis. In fact, a tumor can't easily grow beyond 1 mm in diameter until it has a new blood supply. In the absence of these vessels, cells in the center of the tumor, starved for oxygen and polluted by metabolic waste, die and the tissue becomes necrotic [48]. Although metastatic tumor cells express the genes required to elicit new blood vessel growth, they execute this process in a disorganized manner. In contrast to the predictable and efficient crossroads supplying blood to normal tissues, tumor capillary beds are a tangle of unexpected intersections delivering red blood cells in uneven amounts (Figure 5). The result is often a mass of cells that are rapidly expanding on the periphery but slowly dying in the center. Tumor angiogenesis is therefore a major focus of cancer treatment. In the absence of growing metastatic cells, most cancers could be either cured or treated as a chronic condition. Possession of a well-described model system from which treatment options and predicted responses could be derived might greatly speed progress.

Sprawl development has a consistent complaint: traffic. The root cause of this is simply that sprawl-styled growth forces nearly all travel to be by vehicle. For example, American suburban residents average 13 car trips a day per household, more than twice the number generated by households in traditional neighborhoods [30], p.22). However, the increase in car trips is not the sole source of the problem. While traditional neighborhood designs employ a street grid pattern allowing multiple routes to a given location, this is absent in sprawl developments. In place of a grid, suburban roads are based on a "sparse hierarchy" format, using feeder roads that terminate at a central collector road used by all traffic for short or long distance destinations (Figure 5). This forced travel by automobile has generated some striking statistics. For example, two-thirds of the land area in Los Angeles, CA is dedicated to driving or parking, while Houston, TX possesses nearly 30 paved parking spaces for every resident [40], p.64). Clearly suburban areas employ a form of road design that, like tumor angiogenesis, is resource intensive and inefficient.

To deal with traffic, states such as New Jersey, Maryland and California have adopted context-sensitive road design policies that consider the impact of design decisions on abutting land uses, community character, and the comfort and convenience of pedestrians, cyclists, and transit users as well as automobiles. The utilized techniques include more rigorous management of traffic speeds, narrowing the traffic realm while expanding the pedestrian realm, accommodating bicycles and transit, using curb parking as a buffer between moving traffic and sidewalk activities, and improving the sidewalk environment and ability of pedestrians to cross the street [49]. In contrast, traffic engineers are aware of the adage that dealing with traffic by building more roads is like "trying to cure obesity by loosening your belt". In fact, new roads have consistently been shown to exacerbate traffic problems ([30], pp.88-94). There appears to be a suppressed demand for travel made evident when new roads temporarily improve traffic conditions. Some believe that this "latent demand" is perhaps 30% of the current traffic levels ([41], p122). In contrast, dismantling an existing road predictably reduces local traffic, but surprisingly has little effect on nearby roads ([30], p90). With experience in modifying road design, it seems that traffic engineers might have a lot to say to scientists designing tumor angiogenesis therapies. For example, one could postulate that the existing physiological limits on tumor angiogenesis suppress further tumor growth and spread (perhaps by 30%?), and that cutting off tumor blood vessels will eventually prove to be a useful treatment method without major side effects.

### Similar Causes of Death

One of the striking aspects of cancer is that death is often not directly attributed to tumor-mediated organ failure. Rather, many cancer victims succumb to secondary problems created by the cancer or attempts to cure it. Many late-stage cancer patients develop a wasting syndrome called cachexia that is still poorly understood. During these last weeks or months of life, the body favors energy-burning catabolic reactions over tissue-building anabolic ones. Caloric intake fails to sustain either adipose tissue or lean body mass [50]. The immune system becomes less functional, leaving the patient susceptible to infection. Both of these issues can be aggravated by chemotherapy, which often destroys the intestinal lining and kills white blood cells. Worse yet, cachexia reduces the effectiveness of chemotherapy [51,52]. Approximately 80% of advanced cancer patients develop cachexia [50], which is the direct cause of death in 20-40% of all cancer patients [53-55]. Opportunistic infections often co-occur with cachexia in the advanced stages of cancer. These are deemed the most common cause of preventable death among cancer patients [56]. The precise proportion of cancer patients dying directly from infection varies with cancer type, but several studies place this in the range of 13-45% [57-59]. In the end, patients enter a slow spiral of malnutrition and chronic infection with opportunistic pathogens. Cachexia and the related problems in late stage cancer are perhaps the biggest frustration for both patient and physician. As long as quality of life can be maintained, patients and their caregivers hold onto a hope for improvement and recovery. A better understanding of this wasting syndrome and how to prevent or reverse it could significantly impact cancer therapy.

One consequence of sprawl-styled growth has striking similarities to cachexia. While suburban neighborhoods blossom, older areas nearby often fall into a tenacious "urban blight" zone. This term is somewhat outdated, since older suburbs that have never been truly urban now suffer the same fate as sprawl moves outward away from the city center. The symptoms of blight are obvious: stretches of under-inhabited, poorly cared-for houses near defunct businesses in an area with too little infrastructural support. There seems to be a series of predictable steps in this process. Beginning with departure of inhabitants more rapid than their replacement, the area's tax base is reduced. Lower property values promote further decay and crime (or the perception of it) increases in these areas, perhaps in part because the reduced population offers less surveillance. These factors exacerbate population loss, and decay accelerates. The loss of residents is analogous to healthy functional cells destroyed by tumor impingement and invasion, or as a consequence of chemotherapy. Crime is certainly analogous to the opportunistic pathogens plaguing immunocompromised patients. Perhaps an understanding of urban blight and attempts to reverse it could serve as a model for interventions in cachexia.

Urban blight has been challenged in a variety of ways with some success (as reviewed in detail in [21] pp.153-182). During the 1970's there were attempts to battle this problem by modeling the city after suburbs. Uninhabited houses were torn down to make way for more surface parking, while zoning codes expanded street curb set-back distances and limited on-street parking. Single-use retail shopping malls were built in the city centers. These attempts to re-make cities into suburbs were most often unmitigated failures. Instead of returning to shop or live, the majority of the population found the new design less appealing, more isolating, and more crime-prone. By contrast, cities that have promoted a diversified street life and held onto their time-tested designs have generally faired better. It appears that a critical mass of residential, retail, entertainment, and occupational uses within a mile of the city center provides sufficient support to suppress urban blight. This diversity of uses is analogous to the parenchymal, stromal, and vascular tissues that comprise a healthy organ. Understanding how each element of urban development contributes to survival of the overall structure could be insightful.

A few specific examples of "what works" are instructive. Many neighborhoods have noted and addressed the so-called "broken window" syndrome, the nature of blight described by Wilson and Kelling [60]. Police and others have noted that areas suffering from the early signs of decay - broken windows, graffiti, ill-kept houses - will accelerate into full-blown blight without intervention. The best example of urban decay is perhaps the Pruitt-Igoe urban housing project in St. Louis, Missouri. Shortly after its completion in 1955, the living conditions in Pruitt-Igoe began to decay due to architectural failure, economic decline of St. Louis, white flight into suburbs, and politicized local opposition to government housing projects. By the late 1960s, the extreme poverty, crime, and segregation brought the complex a great deal of infamy. Within 2 decades of its construction, this entire complex of 33 structures was razed and ceased to exist. In contrast, early intervention greatly reduces decay. Urban renewal projects, if properly managed, can yield benefits to the targeted communities. For example, during Quarter 2 of 2009 alone, the 95 active urban renewal projects in City of Los Angeles are expected to create 64,932 construction jobs and 17,115 permanent jobs throughout the City [61].

Many localities now place a premium on early intervention, stopping graffiti and enforcing laws designed to maintain the appearance of homes. There is scientific reason to believe that these efforts can be successful in slowing urban decay. Recent work demonstrates that the surroundings in which people find themselves can significantly alter behavior. In degraded surroundings, subjects were more apt to steal and twice as likely to litter [62]. These data support the idea that urban decay can be self-perpetuating and suggest that early interventions may be productive.

### Predictions from Comparisons of Sprawl and Cancer Systems

While our main objective in this work is to introduce the concept of suburban sprawl and cancer as comparative models for a systems approach to invasive biology, some predictive partners warrant noting (Table 2). The analogous initiating stimuli of financial speculation and aberrant growth factors suggest that just as loss of financial support may slow sprawl, growth factor blockade inhibits cancer. This is already apparent from the use of tamoxifen and herceptin [63,64]. Gasoline costs and p53 function appear to be analogs. Hence our model predicts that if rising fuel costs mitigate sprawl, restoring p53 function will slow cancer spread. Highway construction and angiogenesis are proposed as analogous factors, supporting a comparison of limited highway construction in suburban areas (as proposed in several localities) to the efficacy of anti-angiogenic factors.

**Table 2:** Specific Analogous Factors of Invasive Behavior

Cancer	Sprawl
Fractal growth pattern limited by natural barriers.	Fractal growth pattern limited by natural and man-made barriers

Initiation by growth factors and altered cell signaling	Initiated by profit-based speculative development

Loss of p53 function	Low cost gasoline

Loss of cellular differentiation, expansion of clonal populations	Segregated land use and loss of differentiated architecture

Critical need for angiogenesis, often conducted poorly by tumors	Powerful effects of road development, often constructed in an inefficient manner

Cachexia and Infection	Urban blight

**Figure 6:**
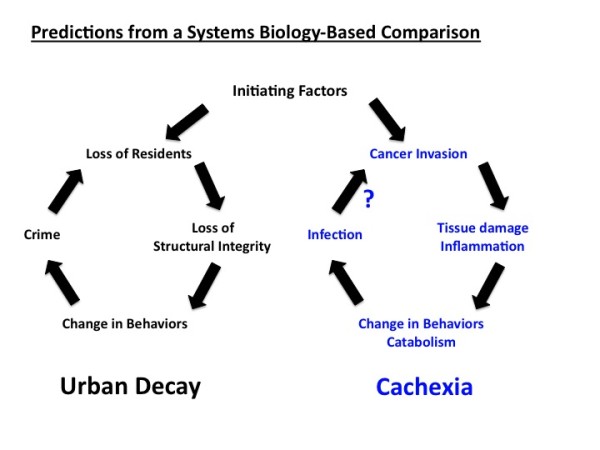
**Predictions Based on Analogous Relationship of Urban Blight and Cachexia**. Left side shows an established pattern involved in urban blight. Right side shows a predicted set of analogously interacting partners promoting cachexia, which are postulated to enhance cancer invasiveness.

While these comparison sets offer logical predictions between the two systems, it is in the areas we least understand that we may gain the most from an analogous system where progress has been made. To this end, in Figure 6 we offer one view of how cancer cachexia and urban blight could be a relevant comparison set. Urban blight is understood as a collection of conditions that are self-propagating, as the loss of residents results in community degradation and crime that furthers the loss of residents. When applied to cachexia, cancer invasion is seen as destabilizing to metabolic homeostasis and immune functioning in normal tissues, resulting in conditions favoring infection. Our model predicts that these conditions are not just by-products of metastasis but actually propagate cancer spread.

Attempts to treat urban blight have potentially important analogies to cachexia. First, interventions to prevent the metabolic shift towards catabolic reactions may be very effective, as they should impact an early stage of this progressive cascade. Improved immune function offers obvious patient benefit - but much more needs to be understood about this process. For example, there is evidence that activated immune cells secreting inflammatory mediators such as tumor necrosis factor may promote cachexia [65]. How can opportunistic infection be averted without a non-protective immune response that worsens the patient's health? More importantly, the proposed model predicts that the behavior of immune and perhaps stromal cells in a cachexic environment may resemble people in blighted surroundings. Studies show these people are more likely to contribute to urban decay, for example by stealing or littering [62]. By analogy, our model predicts that immune and stromal cells from cachexic patients behave in aberrant ways that propagate metabolic decay - perhaps through chronic production of cytokines or other factors. Further, while this aberrant cellular response contributes to opportunistic infection, it may also be reversed if metabolic decay and its fomenting factors are removed. As a specific example, the model predicts that if TNF is an early part of the wasting cascade, anti-TNF therapy may slow the progression of cachexia and subsequently suppress both infection and cancer spread. A detailed understanding of solutions for urban blight may be instructive when examined from a systems biology perspective.

## Implications of the Hypothesis

The cancer-related survival rate has improved modestly from 50% to 66% in 3 decades, but gains in cancers of the pancreas, liver, and ovaries have not been made [1]. Cancer treatment warrants a unique approach. Modeling complex behaviors through mathematical means or by comparisons to analogous systems is a productive way of making progress in challenging areas of science. Since localized cancer has a much lower death rate than disseminated disease, invasiveness is perhaps the most important aspect needing insight. If scale-invariant laws can be used to predict behaviors of similar systems, a well-understood model of invasive behavior may prove fruitful in the development of new anti-tumor therapies. For models to be useful, structure and behavior should be analogous, despite large variations in size. Cancer metastasis and sprawl development represent an ideal analogous pair. In outward appearance, cancer and sprawl exhibit invasive structures that fit fractal geometries with similar mathematical descriptions. More importantly, the initiating factors driving sprawl, its "clonal" expansion, use of poorly constructed "support vessels", and even its mode of "killing" are strikingly similar to metastatic cancer. But unlike potential biological models, suburban sprawl behaves in a way that adheres to man-made laws that led to its development and enforce its continued spread. This offers a tremendous opportunity for modeling behaviors that could lead to new insights for cancer therapy. Furthermore, the development of this model will benefit from interest in halting sprawl. These efforts to redesign zoning laws, limit growth, and revitalize urban centers should provide direct comparisons for efforts to stop tumor metastasis.

We suggest that this analogous pair be further developed to define specific scale-invariant laws of invasive behavior. As shown in Table 2, there are at least 6 specific analogous pairs that can be examined for similarities in these systems. We look forward to in-depth analysis of each of these factors and others that may be apparent upon further examination. A systems biology approach to cancer invasiveness is a logical means of providing insight into this challenging health problem. We hope that this initial description of a tool ready for use leads to further work in this area.

## Competing interests

The authors declare that they have no competing interests.

## Authors' contributions

All authors contributed to the development and testing of this hypothesis. JJR wrote the document with editorial assistance from all authors.
